# The effects of physical activity on mental health in adolescents with attention-deficit hyperactivity disorder: a randomized controlled trial

**DOI:** 10.1186/s12966-025-01745-4

**Published:** 2025-04-17

**Authors:** Chang Liu, Yijian Yang, Stephen Heung-sang Wong, Andes Leung, Cindy Hui-ping Sit

**Affiliations:** 1 Department of Sports Science and Physical Education, The Chinese University of Hong Kong, New Territories, Hong Kong, 00852 China; 2 Vanke School of Public Health, Tsinghua University, Beijing, 100084 China; 3 Runourcity Foundation Limited, Hong Kong, 00852 China

**Keywords:** Adolescent, Aerobic exercise, Attention-deficit hyperactivity disorder, Cognitive function, Depression, Mental health, Resilience

## Abstract

**Background:**

Adolescents with attention-deficit hyperactivity disorder (ADHD) represent a high-risk population with an elevated likelihood of developing mental health disorders. Physical activity (PA) has emerged as a promising intervention to enhance mental health in youth. However, no studies to date have comprehensively examined the immediate and sustained effects of PA, especially aerobic exercise-based PA, on mental ill-being—including internalizing problems (e.g., depression, anxiety, and stress) and externalizing problems (e.g., aggression)—as well as on two other critical indicators of mental health: psychological well-being (e.g., resilience) and cognitive function (e.g., inhibitory control) in adolescents with ADHD. Therefore, this study aimed to investigate whether an aerobic exercise-based PA intervention could elicit immediate and sustained benefits for mental health outcomes, including internalizing problems, externalizing problems, psychological well-being, and cognitive function, in adolescents with ADHD.

**Method:**

This study was an assessor-masked, multicenter, randomized clinical trial. A total of 88 adolescents with ADHD were enrolled. Eligible participants were randomized in a 1:1 ratio to either the exercise group or the control group. Participants in the exercise group attended a 60-min session of aerobic exercise once a week for 12 weeks. Depression, anxiety, stress, aggression, and resilience were assessed using self-report questionnaires, and inhibitory control was evaluated through computer-based neurocognitive tasks. Assessments were conducted at baseline (T0), at the end of the intervention (T1), and 3 months following the intervention (T2).

**Results:**

The 80 eligible participants included 72 (90%) males with a mean age of 14.74 (± 1.59) years. Generalized estimating equation analyses revealed that the current PA intervention resulted in significantly better and sustained improvements in depression, anxiety, stress, and inhibitory control. Compared to the control group, the exercise group showed a significant increase in resilience at T1, but this effect was not sustained at T2. No significant reduction in aggression was found.

**Conclusions:**

The current aerobic exercise-based PA intervention was found to be effective in reducing depression, anxiety, and stress, as well as in promoting inhibitory control and resilience in adolescents with ADHD. The current findings suggest that an aerobic exercise-based PA intervention may be an alternative or adjunctive approach to enhancing mental health, particularly in alleviating internalizing problems, in this population.

**Trial registration:**

ChiCTR, ChiCTR2400087025. Registered 17 July 2024—Retrospectively registered, https://www.chictr.org.cn/showproj.html?proj=230614.

**Supplementary Information:**

The online version contains supplementary material available at 10.1186/s12966-025-01745-4.

## Background

Attention-deficit hyperactivity disorder (ADHD) is a prevalent neurodevelopmental disorder among children and adolescents, with a global prevalence rate of 5.26% [1]. Clinically, ADHD is characterized by patterns of inattention, hyperactivity, and impulsivity [2], and is frequently associated with challenges in emotional regulation, behavioral profiles, and cognitive functioning [3]. Typically emerging in childhood, ADHD often persists into adolescence and adulthood [4, 5]. Adolescents with persistent ADHD are particularly vulnerable to comorbid mental disorders, such as anxiety and conduct disorders [5]. Given that adolescence is a transitional stage with an increased risk of mental health disorders [6], the mental health of adolescents with ADHD warrants greater attention.

Mental ill-being generally encompasses internalizing and externalizing problems [7]. Depression and anxiety are common internalizing problems among children and adolescents with ADHD, occurring at rates of 13.2% and 6.1% [8], respectively, compared to 6.2% and 3.2% in typically developing peers [9]. Comorbid depression and anxiety may lead to long-term impairment [10], and can even result in suicidal behavior [11]. Individuals with ADHD and depression have a fivefold higher rate of suicidal behavior than those with ADHD alone [11]. Elevated perceived stress is also common in adolescents with ADHD and is associated with other internalizing and externalizing problems [12]. Aggression, often observed as a comorbidity of ADHD in children and adolescents, is linked to the persistence of ADHD, serious lifelong functional deficits, and adult antisocial behavior [13]. The presence of comorbid mental ill-being can significantly impact the presentation, treatment, and prognosis of ADHD in adolescents. Addressing mental ill-being, including depression, anxiety, stress, and aggression, in adolescents with ADHD is an urgent priority.

Promoting mental health involves not only the absence of mental ill-being but also the presence of psychological well-being [14]. Resilience is a key indicator of psychological well-being in youth and is considered central to fostering mental health [15]. Defined as a dynamic developmental process that enables adaptation and overcoming negative outcomes, resilience is a protective factor against depression and anxiety in early adulthood for adolescents with ADHD [16]. Mental health, encompassing both well-being and ill-being, is also contingent upon cognitive function, which is a fundamental cornerstone [17]. Lubans et al. [7] have included cognitive function in their conceptual model as a key determinant of mental health for youth, alongside psychological well-being and mental ill-being (i.e., internalizing and externalizing problems). In ADHD, the impairment of inhibitory control is recognized as a core cognitive deficit and is associated with psychopathology in this population [18].

Physical activity (PA) has emerged as a promising intervention for enhancing mental health in children and adolescents with typical development. It encompasses any movement generated by skeletal muscles, characterized by its type, frequency, intensity, duration, and the context in which it occurs [19]. Exercise is a specific subset within the broader category of PA [19]. Meta-analytical research indicates that PA can serve as an alternative or complementary strategy for addressing internalizing and externalizing problems, psychological well-being, and cognitive function in children and adolescents with various neurodevelopmental disorders [20]. Focusing specifically on ADHD, the positive impacts of PA on depression, anxiety, and inhibitory control in children and adolescents have been noted, particularly with non-aerobic exercises such as cognitively engaging activities and primarily within the child population [21, 22]. A study implementing a 12-week combined aerobic and cognitively engaging exercise intervention found that PA had a beneficial effect on inhibitory control, and these effects were sustained for at least 12 weeks following the intervention in children with ADHD [23]. A meta-analysis confirmed the antidepressant benefits of PA for children and adolescents, but no long-term association between PA interventions and depressive symptoms was observed at follow-up periods ranging from 6 to 48 weeks, with no studies specifically targeting children or adolescents with ADHD [24]. Regarding aggression, PA's effect size was found to be non-significant based on studies involving table tennis and horseback riding [25]. Furthermore, no research to date has investigated the impact of PA on resilience in children and adolescents with ADHD [26], despite the proposed and discussed role of PA in fostering resilience during adolescence [27].

Hence, four gaps in the literature were identified: 1) a lack of focus on the effects of PA in adolescents with ADHD; 2) no examination of PA’s effects on the comprehensive aspects of mental health in adolescents with ADHD; 3) the underutilization of aerobic exercise as an intervention; and 4) the limited investigation of the sustained effects of PA on mental health in this population.

To optimize therapeutic change more effectively, it may be beneficial to enhance the mechanisms by which treatment influences outcomes [28]. There are three potential mechanisms that associate PA with mental health in children and adolescents with neurodevelopmental disorders: neurobiological (involving theta activity and P3 amplitude), psychosocial (including social skills and social participation), and behavioral (such as motor skills and sleep) [29]. To further refine the impact of PA on mental health, Vella et al. suggest that PA should be implemented in a manner that: 1) integrates elements that promote adherence and enjoyment; 2) meets individuals'basic needs for autonomy, competence, and social connection; 3) is undertaken in the presence of others who encourage positive interactions and convey a sense of value; 4) occurs outdoors in pleasant natural settings; and 5) is engaged in during leisure time or through active commuting [30].

Consequently, the current study implemented a comprehensive aerobic exercise-based PA intervention designed not only to maximize the potential mechanisms linking PA to mental health but also to adhere to the guidelines proposed by Vella et al. [30]. The aim was to determine if this intervention could elicit immediate and enduring positive effects on mental health outcomes in adolescents with ADHD, encompassing internalizing problems (such as depression, anxiety, and stress), externalizing problems (such as aggression), psychological well-being (particularly resilience), and cognitive function (specifically inhibitory control). The hypothesis posited that aerobic exercise-based PA would provide immediate and sustained benefits for reducing internalizing problems (depression, anxiety, and stress), externalizing problems (aggression), and cognitive deficits (inhibitory control), as well as enhancing psychological well-being (resilience) in adolescents with ADHD.

## Methods

### Study design

This study was conducted as an assessor-masked, multicenter, randomized clinical trial. The setting included both school-based locations and community-based recreational facilities. The trial adhered to the Consolidated Standards of Reporting Trials Extension (CONSORT Extension) for reporting guidelines. Written informed consent was obtained from both participants and their parents prior to the commencement of the study. The research protocol was reviewed and approved by the Joint Chinese University of Hong Kong-New Territories East Cluster Clinical Research Ethics Committee (Reference No.: 2021.645) on December 10, 2022.

### Participants

Participants were recruited from five secondary schools in Hong Kong. Participants were eligible for inclusion if they were aged 12–17, had been clinically diagnosed with ADHD as reported by their parents, and were capable of understanding and completing the questionnaires and computer tests. Exclusion criteria included: (1) medication use (e.g., antidepressants), (2) with other health problems (e.g., asthma disease) that might hinder their PA involvement, and (3) concurrent participation in another behavioral or exercise intervention trial.

### Randomization and masking

Participants were randomly allocated to exercise or control groups at a 1:1 ratio through a computer-based permuted block randomization by an independent research assistant. The allocation was concealed until the intervention was assigned. Assessments were conducted at baseline (T0), the end of the intervention (T1), and 3 months after the intervention (T2) in the classes. The exercise instructors, and the trained outcome assessors were masked to the group allocation.

### Sample size estimation

Estimation of sample size was determined by using G*Power 3.1.9.7. The test family and statistical test were set as ‘F tests’ and ‘ANOVA: Repeated measures, within-between interaction’. By employing an effect size of PA-induced effects on internalizing problems in children and adolescents with neurodevelopmental disorders (Hedges’ g = 0.72) [20], 22 participants were needed to achieve an 80% statistical power (α = 0.05). Assuming a 20% dropout rate, a total sample of at least 28 participants were recruited.

### Intervention

In line with Vella et al.'s guidelines [30], the PA intervention should be implemented during leisure periods. Consequently, a weekly session on weekends would be suitable for secondary school students. Participants in the exercise group attended a 60-min PA session over 12 weeks. Each session was structured into three parts: a 10-min warm-up, a 40-min aerobic exercise, and a 10-min cool-down. The program was designed to be progressive and stepwise in both content and intensity.

During the first six sessions, the interventions took place on school grounds and included enjoyable, less physically demanding activities and mini games (e.g., complex hopscotch and obstacle running). These activities were aimed at gradually improving participants'fitness and motor skills, such as balance, plyometrics, and coordination. These sessions established the physical foundation necessary for the subsequent six sessions. In the final six sessions, running activities were conducted on local streets across various neighborhoods, requiring participants to navigate diverse road networks and utilize greater motor skills. Families of participants were invited to join certain sessions, and some running activities incorporated a volunteering component, such as delivering gifts to elderly individuals. Additionally, sessions were organized where participants ran alongside peers without ADHD. These activities were designed to foster a sense of social belonging and expose participants to different localities, landmarks, and sights.

Previous meta-analyses indicate that intensity does not moderate the effects of PA on internalizing and externalizing problems, cognitive function or psychological well-being in children and adolescents with neurodevelopmental disorders [20]. However, as Vella et al. suggest [30], incorporating elements that enhance adherence, enjoyment, and address individuals'fundamental psychological needs—such as autonomy and competence—can optimize the mental health benefits of PA. Therefore, this study adopted a progressive intensity model, starting with light to moderate intensity during the first six sessions and increasing to moderate to vigorous intensity in the final six sessions. This approach aimed to balance effectiveness with engagement and adherence. Exercise intensity was promoted by a trained coach certified by a non-profit running organization established over a decade ago. The coach observed both participants’ biological and physical status to ensure they reached the desired intensity. Each session was led by a trained coach, assisted by at least one teacher and one research assistant, maintaining an instructor-to-participant ratio of approximately 1:3.

Apart from the PA intervention, participants in the exercise group were instructed to maintain their usual daily routines and were prohibited from participating in any other behavioral or exercise trials. In contrast, participants in the control group did not receive any intervention and continued with their normal daily activities.

### Outcome measure

The primary outcomes focused on mental ill-being, encompassing both internalizing and externalizing problems, including depression, anxiety, stress, and aggression. Depression, anxiety, and stress were measured using the Chinese version of the Depression Anxiety Stress Scale- 21 (DASS- 21) [31]. Each subscale consists of 7 items, rated on a four-point Likert scale (0 = did not apply to me at all, 3 = applied to me very much), with higher scores indicating greater severity of depression, anxiety, or stress. Aggression was assessed using the Chinese version of the Aggression Questionnaire, which includes 12 items rated on a five-point Likert scale ranging from 1 (extremely uncharacteristic of me) to 5 (extremely characteristic of me) [32]. Higher scores on this scale reflect higher levels of aggression.

The secondary outcomes were related to psychological well-being and cognitive function, specifically resilience and inhibitory control. Resilience was measured using the Chinese version of the 25-item Connor-Davidson Resilience Scale (CD-RISC- 25) [33]. Participants were asked to reflect on their experiences over the past month and respond to 25 items using a five-point Likert scale (0 = not true at all, 4 = true all the time). Higher scores on this scale indicate greater resilience.

Inhibitory control was evaluated using the Posner test, a validated tool for assessing inhibitory control in children with developmental coordination disorder [34]. The test consisted of 12 practice trials and 80 experimental trials, divided into two consecutive parts of 40 trials each, with a three-minute rest period between parts. Each part included three types of trials presented in random order: (a) 24 valid trials (60%), where the target stimulus (a red star) appeared in the arrow-oriented stimulus box (a white square with a black outline; 5 cm × 5 cm) after a cue (a yellow arrow); (b) 12 invalid trials (30%), where the target appeared in the arrow-opposite stimulus box after the cue; and (c) 4 neutral trials (10%), where the target appeared without a cue but with a black fixation cross. Each trial began with a 3-s countdown, followed by the appearance of two stimulus boxes and a fixation cross at the midpoint. After 1000 ms, the fixation cross was either replaced by the cue or remained unchanged, and the target stimulus appeared 1000 ms later. Participants were required to respond within 3000 ms by pressing the left red button (“N”) or the right blue button (“M”). An 800 ms buffer interval preceded the start of the next trial. The outcome measures were reaction time (in milliseconds) and response accuracy (in percentage). Shorter reaction times and higher accuracy rates indicated better inhibitory control functioning.

### Statistical analysis

Demographic and clinical outcomes were summarized using percentages (%) or means (standard deviation, SD). The intention-to-treat principle was applied throughout the analysis. Generalized estimating equation (GEE) models were utilized to evaluate the differential changes in key determinants of mental health among adolescents with ADHD, including internalizing problems (i.e., depression, anxiety, and stress), externalizing problems (i.e., aggression), psychological well-being (i.e., resilience), and cognitive function (i.e., inhibitory control). The models adopted a first-order autoregressive structure and controlled for covariates such as sex, age, socioeconomic status, body mass index (BMI), and maternal education level, based on a previous study that examined the association between PA and psychiatric symptoms in youth [35]. Treatment effects were determined by examining the main effects of group and time, as well as their interaction effects. When a significant group × time interaction was identified, the changes within each group at T1 and T2 relative to T0 were calculated, and the differences in these changes between groups were analyzed. Missing data were handled using multiple imputation techniques. All statistical analyses were conducted in R (version 4.3.1, R Foundation for Statistical Computing, Vienna, Austria). Statistical tests were two-tailed, with a significance level set at 5%. Additionally, a per-protocol analysis was performed, and the results are presented in the Appendix.

## Results

### Baseline characteristics of participants

**Fig. 1:**
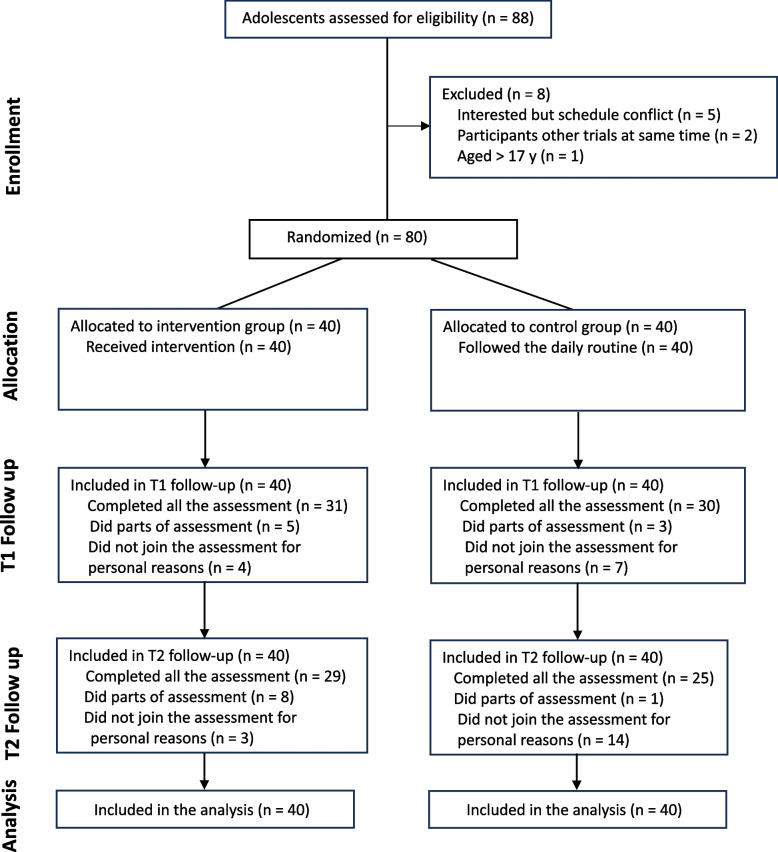
Participants flow diagram

A total of 88 individuals were screened, of whom 8 did not meet the eligibility criteria (Fig. 1). Ultimately, 80 eligible participants were randomly assigned to either the exercise group (35 [88%] male; mean [SD] age, 15.82 [1.11]) or the control group (37 [93%] male; mean [SD] age, 13.65 [1.21]). Participants in the exercise group received at least one session of the intervention, with 36 out of 40 (90%) completing all sessions. At the T1 assessment, the overall dropout rate was 11 out of 80 (14%), with 4 dropouts (10%) in the exercise group and 7 (18%) in the control group. A Chi-Square Test revealed no significant difference in dropout rates between the two groups at T1 (χ^2^ = 0.95, *p* = 0.330). Of the remaining 69 participants, 8 (12%) completed only partial assessments—either the questionnaire or the computer test (Exercise: 5 of 36 [14%]; Control: 3 of 33 [9%]). By the T2 assessment, the overall dropout rate increased to 17 out of 80 (21%), with 3 dropouts (8%) in the exercise group and 14 (35%) in the control group. A Chi-Square Test indicated a significant difference in dropout rates between the two groups at T2 (χ^2^ = 9.04, *p* < 0.01). Among the remaining 63 participants, 9 (14%) completed only partial assessments—either the questionnaire or the computer test (Exercise: 8 of 37 [22%]; Control: 1 of 26 [4%]). All dropouts were attributed to personal reasons unrelated to the trial, and all 80 participants were included in the analyses. The baseline characteristics of the participants are summarized in Table 1.

**Table 1:** Baseline sociodemographic characteristics of the participants

Characteristic			All (*N* = 80)	Group					
				Exercise (*n* = 40)		Control (*n* = 40)		*p*	
Age, mean (SD), y			14.74 (1.59)	15.82 (1.11)		13.65 (1.21)		<.01	
Sex, No. (%)									
Male			72 (90.0%)	35 (87.5%)		37 (92.5%)		.46	
Female			8 (10.0%)	5 (12.5%)		3 (7.5%)			
Body mass index (BMI), mean (SD)			20.91 (3.94)	22.14 (3.98)		19.67 (3.53)		<.01	
Socioeconomic status, No. (%)									
HK$10,000 or less			10 (12.5%)	7 (17.5%)		3 (7.5%)		.02	
HK$10,001–20,000			26 (32.5%)	7 (17.5%)		19 (47.5%)			
Hk$20,001–30,000			18 (22.5%)	9 (22.5%)		9 (22.5%)			
Hk$30,001–40,000			7 (8.8%)	3 (7.5%)		4 (10.0%)			
HK$40,001–50,000			1 (1.3%)	1 (2.5%)		0			
HK$50,001–60,000			6 (7.5%)	6 (15.0%)		0			
HK$60,001–70,000			2 (2.5%)	2 (5.0%)		0			
More than HK$70,000			10 (12.5)	5 (12.5%)		5 (12.5%)			
Maternal education level, No. (%)								.84	
Primary school			13 (16.2%)	5 (12.5%)		8 (20.0%)			
Junior school			19 (23.8%)	11 (27.5%)		8 (20.0%)			
High school			26 (3.25%)	13 (32.5%)		13 (32.5%)			
College-Preparatory			3 (3.8%)	2 (5.0%)		1 (2.5%)			
Non-academic higher education			8 (10.0%)	3 (7.5%)		5 (12.5%)			
Undergraduate education			11 (13.8%)	6 (15.0%)		5 (12.5)			

### Primary outcomes

**Fig. 2:**
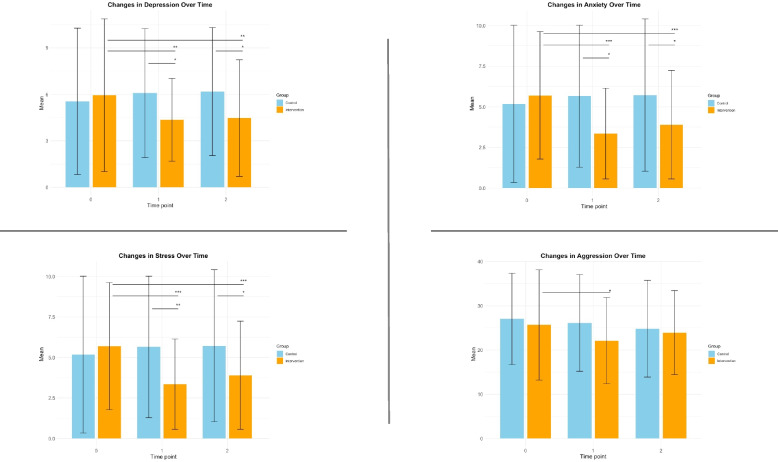
The changes in primary outcomes

The changes in primary outcomes are presented in Fig. 2. Significant interaction effects were observed for depression, anxiety, and stress, but not for aggression (Table 2). In the exercise group, significant reductions were observed in depression, anxiety, and stress at both T1 and T2. Specifically, at T1, the mean changes were as follows: depression (mean change: − 1.60, 95% CI [− 2.73, − 0.47], *p* = 0.005, SMD = − 0.41), anxiety (mean change: − 2.35, 95% CI [− 3.33, − 1.37], *p* < 0.001, SMD = − 0.63), and stress (mean change: − 3.23, 95% CI [− 4.11, − 2.34], *p* < 0.001, SMD = − 0.96). Similarly, at T2, the mean changes were: depression (mean change: − 1.48, 95% CI [− 2.40, − 0.55], *p* = 0.002, SMD = − 0.38), anxiety (mean change: − 1.80, 95% CI [− 2.76, − 0.84], *p* < 0.001, SMD = − 0.48), and stress (mean change: − 2.58, 95% CI [− 3.38, − 1.77], *p* < 0.001, SMD = − 0.77) (Table 3). Compared to the control group, the exercise group demonstrated significantly greater reductions in depression, anxiety, and stress at both T1 and T2. For depression, the adjusted additional change differences were: T1 (− 2.13, 95% CI [− 3.39, − 0.86], *p* < 0.001, SMD = − 0.54) and T2 (− 2.10, 95% CI [− 3.23, − 0.97], *p* < 0.001, SMD = − 0.54). For anxiety, the differences were: T1 (− 2.83, 95% CI [− 4.00, − 1.65], *p* < 0.001, SMD = − 0.70) and T2 (− 2.35, 95% CI [− 3.60, − 1.10], *p* < 0.001, SMD = − 0.59). For stress, the differences were: T1 (− 3.00, 95% CI [− 4.22, − 1.79], *p* < 0.001, SMD = − 0.81) and T2 (− 2.08, 95% CI [− 3.37, − 0.78], *p* < 0.001, SMD = − 0.56) (Table 3).

**Table 2:** Summary of generalized estimated equation analysis

Measurement	Mean (SD)		*P* value		
	Exercise	Control	Interaction effect	Group effect	Time effect
**Primary outcomes**					
*** Internalizing problems***					
Depression					
T0	5.95 (4.93)	5.55 (4.73)	< .001	.43	.28
T1	4.35 (2.67)	6.08 (4.17)			
T2	4.47 (3.76)	6.18 (4.13)			
Anxiety					
T0	5.70 (4.66)	5.18 (4.84)	< .001	.39	.02
T1	3.35 (2.95)	5.65 (4.37)			
T2	3.90 (3.43)	5.72 (4.30)			
Stress					
T0	6.60 (3.92)	6.40 (4.62)	< .001	.23	< .001
T1	3.38 (2.79)	6.18 (4.34)			
T2	4.03 (3.34)	5.90 (4.69)			
*** Externalizing problems***					
Aggression					
T0	25.68 (12.47)	27.05 (10.34)	.43	.30	.19
T1	22.12 (9.76)	26.15 (10.95)			
T2	23.95 (9.49)	24.85 (10.97)			
**Secondary outcomes**					
*** Psychological well-being***					
Resilience					
T0	50.85 (24.37)	46.42 (20.27)	.001	.24	.12
T1	56.50 (16.94)	43.90 (17.75)			
T2	47.88 (17.65)	44.98 (20.78)			
*** Cognitive function***					
Inhibitory control					
Valid RT, ms					
T0	447.01 (63.60)	488.29 (339.83)	< .001	.19	.14
T1	399.69 (51.29)	502.35 (352.71)			
T2	405.06 (53.39)	505.65 (358.80)			
Valid ACC, %					
T0	0.97 (0.06)	0.98 (0.06)	.15	.76	.02
T1	0.98 (0.07)	0.94 (0.15)			
T2	0.94 (0.13)	0.95 (0.11)			
Invalid RT, ms					
T0	475.21 (93.93)	510.08 (320.72)	< .001	.25	.21
T1	427.91 (66.85)	527.27 (343.62)			
T2	441.68 (92.44)	520.14 (0.10)			
Invalid ACC, %					
T0	0.97 (0.09)	0.98 (0.08)	.92	.71	.07
T1	0.93 (0.19)	0.95 (0.11)			
T2	0.94 (0.13)	0.94 (0.10)			
Neutral RT, ms					
T0	478.56 (94.17)	526.82 (395.38)	< .001	.14	.17
T1	426.79 (67.57)	541.13 (325.26)			
T2	419.73 (53.92)	550.76 (390.45)			
Neutral ACC, %					
T0	0.97 (0.11)	0.99 (0.03)	.83	.40	.06
T1	0.94 (0.16)	0.94 (0.16)			
T2	0.96 (0.12)	0.97 (0.05)			

*ACC* accuracy, *RT* reaction time

**Table 3:** Summary of post hoc analysis of generalized estimated equation analysis

Measurement	Exercise			Control			Exercise—Control		
	Mean(95% CI)	*P* value	SMD	Mean(95% CI)	*P* value	SMD	Adjusted additional change difference (95% CI)	*P* value	SMD
**Primary outcomes**									
*** Internalizing problems***									
Depression									
T1-T0	− 1.60 (− 2.73 to − 0.47)	.005	− 0.41	0.53 (− 0.05 to 1.10)	.07	0.12	− 2.13 (− 3.39 to − 0.86)	<.001	− 0.54
T2-T0	− 1.48 (− 2.40 to − 0.55)	.002	− 0.38	0.63 (− 0.05 to 1.30)	.07	0.15	− 2.10 (− 3.23 to − 0.97)	<.001	− 0.54
Anxiety									
T1-T0	− 2.35 (− 3.33 to − 1.37)	<.001	− 0.63	0.48 (− 0.19 to 1.14)	.15	0.11	− 2.83 (− 4.00 to − 1.65)	<.001	− 0.70
T2-T0	− 1.80 (− 2.76 to − 0.84)	<.001	− 0.48	0.55 (− 0.26 to 1.36)	.18	0.12	− 2.35 (− 3.60 to − 1.10)	<.001	− 0.59
Stress									
T1-T0	− 3.23 (− 4.11 to − 2.34)	<.001	− 0.96	− 0.23 (− 1.07 to 0.62)	.60	− 0.05	− 3.00 (− 4.22 to − 1.79)	<.001	− 0.81
T2-T0	− 2.58 (− 3.38 to − 1.77)	<.001	− 0.77	− 0.50 (− 1.53 to 0.53)	.33	− 0.11	− 2.08 (− 3.37 to − 0.78)	<.001	− 0.56
**Secondary outcomes**									
*** Psychological well-being***									
Resilience									
T1-T0	5.65 (1.51 to 9.79)	.007	0.29	− 2.52 (− 5.74 to 0.69)	.12	− 0.13	8.18 (2.96 to 13.39)	.002	0.42
T2-T0	− 2.97 (− 9.66 to 3.71)	.38	− 0.15	− 1.45 (− 6.20 to 3.30)	.55	− 0.07	− 1.53 (− 9.68 to 6.30)	.71	− 0.08
*** Cognitive function***									
Inhibitory control									
Valid RT, ms									
T1-T0	− 47.32 (− 68.00 to − 26.6)	<.001	− 0.85	14.1 (− 8.93 to 37.1)	.23	0.04	− 61.39 (− 92.18 to − 30.60)	<.001	− 0.27
T2-T0	− 41.95 (− 62.22 to − 22.00)	<.001	− 0.75	17.4 (− 18.18 to 52.9)	.33	0.05	− 59.32 (− 99.89 to − 18.74)	.004	− 0.26
Invalid RT, ms									
T1-T0	− 47.3 (− 68.4 to − 26.19)	<.001	− 0.56	17.2 (− 5.64 to 40.0)	.14	0.05	− 64.50 (− 95.45 to − 33.55)	<.001	− 0.29
T2-T0	− 33.5 (− 70.3 to 3.19)	.07	− 0.40	10.1 (− 32.00 to 52.1)	.64	0.03	− 43.59 (− 99.15 to 11.96)	.12	− 0.20
Neutral RT, ms									
T1-T0	− 51.8 (− 79.4 to − 27.2)	<.001	− 0.71	14.3 (− 21.01 to 49.7)	.42	0.04	− 66.09 (− 109.00 to − 23.18)	.002	− 0.27
T2-T0	− 58.8 (− 82.2 to − 35.5)	<.001	− 0.80	23.9 (− 7.01 to 54.9)	.13	0.06	− 82.77 (− 121.36 to 44.18)	<.001	− 0.33

*CI* confidence interval, *RT* reaction time, *SMD* standardized mean difference

### Secondary outcomes

Significant interaction effects were observed for resilience and inhibitory control (i.e., reaction time in valid, invalid, and neutral trials) (Table 2). In the exercise group, resilience showed a significant improvement at T1 compared to T0 (mean change: 5.65, 95% CI [1.51, 9.79], *p* = 0.007, SMD = 0.29), but no significant change was observed at T2 (mean change: − 2.97, 95% CI [− 9.66, 3.71], *p* = 0.38) (Table 3). Compared to the control group, the exercise group demonstrated a significant increase in resilience at T1 (adjusted additional change difference: 8.18, 95% CI [2.96, 13.39], *p* = 0.002, SMD = 0.42), but no significant differences were found between the groups at T2 (adjusted additional change difference: − 1.53, 95% CI [− 9.68, 6.30], *p* = 0.71) (Table 3).

Regarding inhibitory control, significant reductions in reaction time were observed in the exercise group for both valid and neutral trials at T1 (valid trials: mean change: − 47.32 ms, 95% CI [− 68.00, − 26.60], *p* < 0.001, SMD = − 0.85; neutral trials: mean change: − 51.80 ms, 95% CI [− 79.40, − 27.20], *p* < 0.001, SMD = − 0.71) and T2 (valid trials: mean change: − 41.95 ms, 95% CI [− 62.22, − 22.00], *p* < 0.001, SMD = − 0.75; neutral trials: mean change: − 58.80 ms, 95% CI [− 82.20, − 35.50], *p* < 0.001, SMD = − 0.80) (Table 3). Compared to the control group, the exercise group showed significant reductions in reaction time for valid and neutral trials at both T1 (valid trials: adjusted additional change difference: − 61.39 ms, 95% CI [− 92.18, − 30.60], *p* < 0.001, SMD = − 0.27; neutral trials: adjusted additional change difference: − 66.09 ms, 95% CI [− 109.00, − 23.18], *p* = 0.002, SMD = − 0.27) and T2 (valid trials: adjusted additional change difference: − 59.32 ms, 95% CI [− 99.89, − 18.74], *p* = 0.004, SMD = − 0.26; neutral trials: adjusted additional change difference: − 82.77 ms, 95% CI [− 121.36, − 44.18], *p* < 0.001, SMD = − 0.33) (Table 3). For invalid trials, reaction time in the exercise group significantly decreased at T1 (mean change: − 47.30 ms, 95% CI [− 68.40, − 26.19], *p* < 0.001, SMD = − 0.56), but no significant change was observed at T2. Similarly, when compared to the control group, the exercise group showed a significant reduction in reaction time for invalid trials at T1 (adjusted additional change difference: − 64.50 ms, 95% CI [− 95.45, − 33.55], *p* < 0.001, SMD = − 0.29), but not at T2.

## Discussion

The current study primarily examined the effects of aerobic exercise-based PA on two critical aspects of mental health in adolescents with ADHD: internalizing problems (i.e., depression, anxiety, and stress) and externalizing problems (i.e., aggression). The findings demonstrated that aerobic exercise-based PA significantly improved depression, anxiety, and stress, but did not show significant effects on aggression. Notably, the positive effects on depression, anxiety, and stress were sustained for three months post-intervention. These primary results were consistent with the per-protocol analysis, as detailed in the Appendix. Additionally, positive effects were observed in psychological well-being (i.e., resilience) and cognitive function (i.e., inhibitory control). However, while the aerobic exercise-based intervention led to sustained improvements in inhibitory control, specifically in valid and neutral trials, no lasting effects were observed for invalid trials or resilience.

Regarding the primary outcomes, the current study did not observe a significant reduction in aggression, a finding consistent with a previous meta-analysis that reported a non-significant effect of PA on aggressive behavior in children and adolescents with ADHD [25]. Another meta-analysis similarly noted a non-significant effect of PA on self-reported aggression in this population, suggesting that the type of informant (e.g., self-report vs. teacher-report) may influence the validity of aggression measures [36]. Previous research has demonstrated that while self-reports and teacher-reported aggression scores are correlated, it is the teachers'reports, rather than the students'self-reports, that significantly predict actual aggressive behaviors [37]. Therefore, future studies are encouraged to employ multiple informants and measures to assess aggressive behavior more comprehensively.

Additionally, the characteristics of PA interventions, such as duration and intensity, may serve as moderators of their effectiveness. A prior meta-analysis found that the duration of each PA session significantly moderated its effects on externalizing problems in children and adolescents with neurodevelopmental disorders [20]. Specifically, 30 min per session was identified as the optimal duration, while longer sessions (30–90 min) reduced the effectiveness of PA [20]. In the current study, the 60-min session duration may have been excessive for adolescents with ADHD, potentially leading to fatigue and irritability [38]. Furthermore, unlike the current study, which adopted a progressive PA intensity, and other studies that either did not report intensity or found no significant effects of PA on aggression in this population [39–41], one earlier study demonstrated a significant reduction in aggression when PA was consistently conducted at moderate-to-vigorous intensity [42]. This suggests that intensity may be a critical factor influencing the effects of PA on behavioral outcomes. For instance, increasing the intensity of PA has been shown to enhance self-control, which may further contribute to reducing aggressive behavior [43]. Thus, future research should consider optimizing both the duration and intensity of PA interventions to maximize their benefits for reducing aggression in individuals with ADHD.

However, the current exercise intervention demonstrated greater benefits for internalizing problems, including depression, anxiety, and stress, which contrasts with findings from previous experimental studies. Three prior studies investigating the effects of PA on depression and anxiety in children with ADHD reported non-significant effects [39, 41, 42]. The discrepancies between these studies and the current findings may be attributed to three key factors. First, the current intervention employed aerobic exercise, whereas the previous studies utilized cognitively engaging exercises (e.g., table tennis, ball games, and hippotherapy). Meta-analytical evidence suggests that aerobic exercise (e.g., running and swimming) may be more effective than cognitively engaging exercises in alleviating internalizing problems among children and adolescents with neurodevelopmental disorders [20]. The antidepressant effects of aerobic exercise may be explained by the stimulation of endorphins and the increase in neurotransmitters such as serotonin and dopamine [44]. Similarly, aerobic exercise is thought to reduce anxiety by generating bodily sensations similar to those that trigger anxious responses, thereby diminishing fear and altering the interpretation of such stimuli through repeated exposure [45]. Additionally, while these three previous studies focused on children with ADHD [39, 41, 42], the current study targeted adolescents. Adolescence is characterized by greater histological and chemical brain maturity compared to childhood [44], which may significantly influence mental and psychological states [46]. Furthermore, the current study was conducted during leisure time across multiple centers. The context in which PA is undertaken plays a crucial role [47], as evidenced by a previous meta-analysis showing that promoting PA during leisure time is likely the most effective approach to preventing mental ill-being compared to other PA domains [48].

Regarding stress, only one prior study examined the effects of PA on stress in children and adolescents with ADHD and confirmed its beneficial effects [49]. This study also utilized aerobic exercise, specifically swimming training. Meta-analytical findings suggest that aerobic exercise significantly benefits stress-related blood pressure responses [50]. The current intervention further incorporated activities aimed at enhancing participants'motor skills, such as coordination exercises. Both aerobic and coordinative exercises have stress-buffering effects with a neurochemical basis linked to the hypothalamic–pituitary–adrenal axis, including the modulation of cortisol release, a key stress hormone [51, 52]. The hypothalamic–pituitary–adrenal axis is considered the primary neuroendocrine response facilitating adaptation to stress [53]. However, this previous experimental study did not observe significant effects of swimming on anxiety [49], possibly because the current aerobic exercise intervention included social-related factors, such as running on local streets, inviting participants'parents and peers to join, and delivering gifts to the elderly. These activities may have promoted social participation and social skills among participants. Evidence suggests that neural circuits supporting affective processing are highly sensitive to social-contextual influences, even within relatively short time frames or discrete interactions with others [54].

Additionally, the beneficial effects of aerobic exercise on depression, anxiety, and stress were found to persist for three months in the current exercise group. Several factors may explain this sustained impact. First, individuals with lower baseline performance levels may have greater room for improvement due to floor effects [55]. A previous meta-analysis demonstrated that adolescents with clinical diagnoses experienced greater benefits from PA interventions for internalizing problems compared to typically developing peers [56]. Given that adolescents with ADHD frequently experience mental health challenges, they may derive more substantial and longer-lasting benefits from PA interventions targeting depression, anxiety, and stress. Furthermore, physiological adaptations, such as changes in sympathetic nervous system activity and hormonal responses following aerobic exercise, may contribute to the sustainability of these effects [45]. These adaptations are believed to be closely linked to the physical symptoms of anxiety and other mental health conditions, providing a potential mechanism for the long-term benefits observed [45].

Consistent with a previous meta-analysis examining the effects of chronic exercise on inhibitory control [57], the current study also demonstrated positive outcomes. The content of the PA intervention may be a key factor contributing to these results. While the current intervention primarily focused on aerobic exercise, it also incorporated activities aimed at enhancing participants'motor skills, such as balance, plyometrics, and coordination. Both acute and chronic coordinative exercise may stimulate greater prefrontal cortex activity [58, 59], which is closely associated with cognitive control [60]. As the motor demands increased over the course of the intervention, greater prefrontal cortex activity was likely required to perform these tasks effectively [61].

However, while the current intervention yielded sustained improvements in reaction times for valid and neutral trials of inhibitory control, no significant difference was observed for invalid trials at the 3-month follow-up compared to baseline. Invalid trials are more complex than valid and neutral trials, potentially requiring a broader range of frontal-dependent cognitive processes. Intensity may play a critical role in enhancing the beneficial effects of PA on neurocognitive functions [62]. Meta-analytical findings suggest that moderate-intensity PA produces more significant effects on executive function compared to light, moderate-to-vigorous, or vigorous intensity [21]. In contrast, the current study adopted a progressive and stepwise training model, which may not have provided the necessary intensity to fully activate the cognitive processes required for invalid trials. This perspective also helps explain why no significant benefit was observed for aggression in the current study, as previously discussed. Regulating behavior through self-control—a component of inhibitory control—may demand more extensive cognitive processes, and the current intervention might not have been sufficiently intensive to activate the necessary brain regions. Additionally, frequency (i.e., sessions per week) is another critical factor influencing the effects of PA on cognitive function in children and adolescents with neurodevelopmental disorders, with higher frequency associated with larger effect sizes [20]. Frequent and repeated practice may be more effective in enhancing the benefits of PA interventions for this population [63]. Therefore, future research should aim to optimize the content, intensity, and frequency of PA interventions to maximize their benefits for inhibitory control in children and adolescents with ADHD.

A higher frequency of PA may also be necessary due to its contribution to greater effects on the psychological well-being of children and adolescents with neurodevelopmental disorders [20]. The current study only examined the immediate effects of aerobic exercise on resilience, not its sustained effects. The observed PA-induced benefits for resilience align with a previous meta-analysis demonstrating that PA interventions can enhance resilience in children and adolescents [64]. The reduction in negative emotional reactivity and the improvement in cognitive function observed in this study may both contribute to the increase in resilience [65]. However, the adaptation of resilience may further require the coordination of multiple aspects. Fostering resilience likely requires the coordination of multiple systems, as resilience is not merely a personal trait but a dynamic mental process that emerges from the interplay of biological, psychological, social, and ecological systems [66, 67]. The current intervention created an environment that allowed adolescents with ADHD to engage in contexts where multiple systems interact. For example, running on local streets with family or peer companions integrated physical, social, and psychological systems, promoting a holistic approach to resilience. However, sustaining these positive changes is challenging unless other systems are sufficiently robust and resources are adequate to support new resilience-building practices [67]. This is particularly relevant for adolescents with ADHD, who often face impairments across multiple systems, such as family conflicts, strained peer relationships, and chronic health issues [15]. Therefore, while the current PA intervention provided a foundation for resilience, long-term benefits may depend on the strength and stability of these interconnected systems.

The World Health Organization (WHO) recommends that children and adolescents engage in an average of 60 min of moderate to vigorous PA daily to enhance physical, mental, and cognitive health outcomes [68]. However, the current study observed positive effects on internalizing problems (e.g., depression, anxiety, and stress), cognitive function (inhibitory control), and psychological well-being (resilience) despite implementing a 12-week intervention with a progressive intensity of only once per week and 60 min per session. Several factors may explain these findings. First, the intervention enhanced potential mechanisms linking PA to mental health, such as motor skills and social connections, which are often underdeveloped in individuals with ADHD [3]. By addressing these areas, the intervention optimized the mental health benefits of PA. Second, the PA intervention adhered to the recommendations of Vella et al. [30] by: 1) including games and volunteer activities to promote enjoyment; 2) using progressive intensity to promote adherence and address their competence; 3) encouraging participation with their parents and typically developed peers to foster social connection and a sense of value; 4) conducting sessions outdoors in pleasant natural environments; and 5) scheduling sessions during leisure time. These factors likely amplified the positive effects of PA. Third, adolescents with ADHD often experience more pronounced mental health deficits [3], making them more likely to benefit from even limited PA interventions. This suggests that maybe a modest increase in PA could yield significant improvements in mental health for this population.

The current study also offers several practical implications. As shown in Table 2, the effect sizes (SMD) indicate that the aerobic exercise-based PA intervention had a stronger impact on internalizing problems compared to inhibitory control. This finding is consistent with previous meta-analyses, which suggest that aerobic exercise provides greater benefits for internalizing problems than cognitively engaging exercise in children and adolescents with neurodevelopmental disorders [20]. This highlights that aerobic exercise may be the preferred PA intervention for addressing internalizing problems in adolescents with ADHD. However, when it comes to improving cognitive function in this population, more targeted PA interventions may need to be explored and developed.

This study has several limitations. First, the distribution of sexes was uneven, with only eight female participants, potentially introducing bias into the results. Second, the intensity of PA was not objectively optimized or measured using tools such as Polar heart rate monitors, which could have influenced the treatment effects of PA. Third, while the intervention enhanced potential mechanisms linking PA to mental health, such as motor skills and social participation, these mechanisms were not thoroughly examined. Future studies should further investigate the neurobiological, psychosocial, and behavioral mechanisms underlying the relationship between PA and mental health in adolescents with ADHD. Fourth, the dropout rates between the intervention and control groups were significantly different at T2. However, several measures were implemented to mitigate the potential impact of these differing dropout rates. The reasons for participant dropout were documented, revealing that no trial-related factors (e.g., side effects) contributed to the dropout. A per-protocol analysis was conducted, and the results, provided in the Appendix, were consistent with the main findings, suggesting that the dropout rates likely did not affect the intervention outcomes. Additionally, multiple imputation was used to handle missing data, a method considered reliable even when a significant proportion of data is missing [69]. These approaches collectively strengthened the reliability of the study results. Fifth, participants were recruited from five secondary schools in Hong Kong using a convenience sampling method, which may introduce biases into the results. Future research should consider more diverse and representative sampling methods to enhance the generalizability of the findings.

## Conclusions

Among adolescents with ADHD, engaging in aerobic exercise-based PA may yield significant benefits for internalizing problems (i.e., depression, anxiety, and stress), psychological well-being (i.e., resilience), and cognitive function (i.e., inhibitory control). The treatment effects on internalizing problems and cognitive function appeared to be sustained for at least three months in the exercise group. These findings suggest that aerobic exercise-based PA could serve as an alternative or adjunctive approach to promoting mental health, particularly in alleviating depression, anxiety, and stress, in adolescents with ADHD. Future aerobic exercise-based interventions could consider incorporating additional sessions with increased frequency and shorter durations, specifically tailored to this population, to further enhance overall mental health outcomes.

## Supplementary Information

Supplementary Material 1.

## Data Availability

The datasets used and/or analysed during the current study are available on reasonable request.

## Abbreviations

ADHD: adolescents with attention-deficit hyperactivity disorder
PA: physical activity
