# Supplementary material for: The effects of physical activity on mental health in adolescents with attention-deficit hyperactivity disorder: a randomized controlled trial
Source: Int J Behav Nutr Phys Act. 2025 Apr 17;22:47. doi: 10.1186/s12966-025-01745-4 (PMC12007287; doi:10.1186/s12966-025-01745-4)
Supplement: Supplementary file 1 — Supplementary Material 1. [file 12966_2025_1745_MOESM1_ESM.pdf]

The effects of physical activity on mental health in adolescents with attention-deficit hyperactivity disorder: A randomized controlled trial

Chang Liu PhD,[a,b] Yijian Yang PhD,[a] Stephen Heung-sang Wong PhD,[a]
Andes Leung PhD,[c] Cindy Hui-ping Sit PhD [a]

[a] Department of Sports Science and Physical Education, The Chinese University of Hong Kong, Shatin, New Territories, Hong Kong 00852, China

[b] Vanke School of Public Health, Tsinghua University, Beijing 100084, China

[c] RunOurCity Foundation Limited, Hong Kong, China

Address correspondence to: Cindy Hui-ping Sit, Department of Sports Science and Physical Education, The Chinese University of Hong Kong, Shatin, New Territories, Hong Kong, China, sithp@cuhk.edu.hk, +852 3943 4126.

Results for per-protocol analysis

Baseline characteristics of Participants

Overall, 44 participants were included in per-protocol analysis, of which 23 were assigned to exercise (19 [82.6%] male; mean [SD] age, 15.57 [1.20]) and 21 were in control group (20 [95.2%]; mean [SD] age, 13.24 [1.00]). The baseline characteristics of participants are presented in Table 1.

Primary Outcomes

Significant interaction effects were observed for depression, anxiety, and stress, but not for aggression (Table 2). In the exercise group, significant reductions were observed in depression, anxiety, and stress at both T1 and T2. Specifically, at T1, the mean changes were as follows: depression (mean change: -2.57, 95% CI [-3.76, -1.37], $p < .001$, SMD = -0.71), anxiety (mean change: -2.91, 95% CI [-3.94, -1.89], p < .001, SMD = -0.88), and stress (mean change: -3.35, 95% CI [-4.35, -2.35], $p < .001$, SMD = -1.16). Similarly, at T2, the mean changes were: depression (mean change: -2.30, 95% CI [-3.44, -1.17], $p < .001$, SMD = -0.64), anxiety (mean change: -2.65, 95% CI [-3.90, -1.41], $p < .001$, SMD = -0.80), and stress (mean change: -2.83, 95% CI [-3.38, -1.82], $p < .001$, SMD = -0.98) (Table 3). Compared to the control group, the exercise group demonstrated significantly greater reductions in depression, anxiety, and stress at both T1 and T2. For depression, the adjusted additional change differences were: T1 (-2.52, 95% CI [-3.84, -1.19], p < .001, SMD = -0.59) and T2 (-2.59, 95% CI [-3.88, -1.30], $p < .001$, SMD = -0.61). For anxiety, the differences were: T1 (-3.32, 95% CI [-4.56, -2.12], $p < .001$, SMD = -0.77) and T2 (-3.41, 95% CI [-4.82, -2.01], $p < .001$, SMD = -0.79). For stress, the differences were: T1 (-2.44, 95% CI [-3.83, -1.06], $p < .001$, SMD = -0.62) and T2 (-2.45, 95% CI [-4.08, -0.81], $p = .003$, SMD = -0.62) (Table 3).

Secondary outcomes

Significant interaction effects were observed for resilience and inhibitory control (i.e., reaction time in valid, invalid, and neutral trials; accuracy in valid trials) (Table 2). In the exercise group, resilience

showed a significant improvement at T1 compared to T0 (mean change: 7.04, 95% CI [2.86, 11.2], < .001, SMD = 0.38), but no significant change was observed at T2 (mean change: 4.17, 95% CI [-1.94, 10.3], p = .17) (Table 3). Compared to the control group, the exercise group demonstrated a significant increase in resilience at T1 (adjusted additional change difference: 10.14, 95% CI [4.83, 15.45], p < .001, SMD = 0.57), but no significant differences were found between the groups at T2 (adjusted additional change difference: 5.79, 95% CI [-0.86, 12.44], p = .08) (Table 3).

Regarding inhibitory control, significant reductions in reaction time were observed in the exercise group for both valid and neutral trials at T1 (valid trials: mean change: -51,10 ms, 95% CI [-76.00, -26.60], p < .001, SMD = -0.95; Invalid trials: mean change: -51,70 ms, 95% CI [-80.00, -23.36], p < .001, SMD = -0.63; neutral trials: mean change: -48.50 ms, 95% CI [-75.30, -21.70], p < .001, SMD = -0.67) and T2 (valid trials: mean change: -44.60 ms, 95% CI [-62.22, -22.00], p < .001, SMD = -0.83; Invalid trials: mean change: -42.90 ms, 95% CI [-85.00, -0.68], p = .04, SMD = -0.21; neutral trials: mean change: -52.50 ms, 95% CI [-83.70, -21.30], p < .001, SMD = -0.72). Compared to the control group, the exercise group showed significant reductions in reaction time for valid trial and invalid trial at both T1 (valid trials: adjusted additional change difference: -74.60 ms, 95% CI [-109.14, -39.97], p < .001, SMD = -0.26; Invalid trials: adjusted additional change difference: -81.00 ms, 95% CI [-121.98, -40.09], p < .001, SMD = -0.30) and T2 (valid trials: adjusted additional change difference: -92.70 ms, 95% CI [-148.71, -36.71], p = .001, SMD = -0.32; Invalid trials: adjusted additional change difference: -73.50 ms, 95% CI [-135.91, -11.06], p = .02, SMD = -0.27) (Table 3). As for neutral trials, when compared to the control group, the exercise group showed a significant reduction in reaction time at T2 (adjusted additional change difference: -97.83ms, 95% CI [-144.96, -50.71], p < .001, SMD = -0.32), but not at T1. Although there were no significant changes of accuracy for valid trials in exercise at neither T1 nor T2, when compared to the control group, the exercise group showed significant improvements in accuracy for valid trials at T1 (adjusted additional change difference: 0.08 ms, 95% CI [0.02, 0.15], p = .01, SMD = 0.83), but not T2.

Table 1. Baseline Sociodemographic Characteristics of the Participants

Characteristic	All (N = 44)	Group		
		Exercise (n = 23)	Control (n = 21)	*P*
Age, mean (SD), y	14.45 (1.61)	15.57 (1.20)	13.24 (1.00)	<.01
Sex, No. (%)				
Male	39 (88.6%)	19 (82.6%)	20 (95.2%)	.19
Female	5 (11.4%)	4 (17.4%)	1 (4.8%)	
Body mass index (BMI), mean (SD)	20.63 (3.61)	21.70 (3.48)	19.45 (3.45)	.04
Socioeconomic status, No. (%)				
HK$10,000 or less	4 (9.1%)	3 (13.0%)	1 (4.8%)	
HK$10,001-20,000	13 (29.5%)	3 (13.0%)	10 (47.6%)	
Hk$20,001-30,000	13 (29.5%)	7 (30.4%)	6 (28.6%)	
Hk$30,001-40,000	4 (9.1%)	3 (7.5%)	1 (4.8%)	.13
HK$40,001-50,000	0	0	0	
HK$50,001-60,000	3 (6.8%)	3 (13.0%)	0	
HK$60,001-70,000	1 (2.3%)	1 (4.3%)	0	
More than HK$70,000	6 (13.6%)	3 (13.0%)	3 (14.3%)	
Maternal education level, No. (%)				
Primary school	8 (18.2%)	4 (14.7%)	4 (19.0%)	
Junior school	9 (20.5%)	5 (21.7%)	4 (19.0%)	
High school	15 (34.1%)	8 (34.8%)	7 (33.3%)	.94
College-Preparatory	1 (2.3%)	1 (4.3%)	0	
Non-academic higher education	5 (11.4%)	2 (8.7%)	3 (14.3%)	
Undergraduate education	6 (13.6%)	3 (13.0%)	3 (14.3%)	

Table 2. Summary of Generalized Estimated Equation Analysis

Measurement	Mean (SD)		*P* value		
	Exercise	Control	Interaction effect	Group effect	Time effect
**Primary outcomes**					
Internalizing problems					
Depression					
T0	6.91 (4.42)	6.62 (5.71)			
T1	4.35 (2.71)	6.57 (5.28)	< .001	.44	.002
T2	4.61 (3.65)	6.90 (4.98)			
Anxiety					
T0	6.65 (3.98)	5.86 (6.04)			
T1	3.74 (3.02)	6.29 (5.43)	< .001	.46	.003
T2	4.00 (3.02)	6.62 (5.16)			
Stress					
T0	6.74 (3.12)	7.62 (5.47)			
T1	3.39 (2.95)	6.71 (5.30)	< .001	.07	< .001
T2	3.91 (2.71)	7.24 (5.31)			
Externalizing problems					
Aggression					
T0	24.43 (11.75)	29.62 (11.16)			
T1	22.83 (11.61)	29.76 (11.07)	.80	.02	.83
T2	23.61 (8.70)	28.67 (10.16)			
**Secondary outcomes**					
Psychological well-being					
Resilience					
T0	50.09 (22.77)	44.62 (19.98)			
T1	57.13 (16.63)	41.52 (16.83)	.001	.11	.36
T2	54.26 (16.46)	43.00 (19.51)			
Cognitive function					
Inhibitory control					
Valid RT, ms					
T0	440.59 (68.47)	529.39 (458.38)			
T1	389.49 (48.66)	552.85 (481.21)	< .001	.18	.10
T2	395.96 (44.31)	577.47 (485.36)			
Valid ACC, %					
T0	0.97 (0.07)	0.98 (0.08)			
T1	0.99 (0.02)	0.92 (0.21)	.008	.24	.47
T2	0.98 (0.05)	0.94 (0.12)			
Invalid RT, ms					
T0	471.35 (103.64)	550.39 (431.43)			
T1	419.69 (74.06)	579.76 (468.03)	< .001	.21	.52
T2	428.50 (68.73)	581.02 (432.49)			
Invalid ACC, %					
T0	0.96 (0.11)	0.97 (0.10)			
T1	0.95 (0.21)	0.93 (0.15)	.64	.75	.78
T2	0.97 (0.10)	0.94 (0.09)			
Neutral RT, ms					
T0	473.04 (97.49)	583.55 (539.81)			
T1	424.57 (68.26)	585.24 (441.97)	< .001	.16	.29
T2	420.54 (47.62)	628.88 (525.61)			
Neutral ACC, %					
T0	0.95 (0.14)	0.99 (0.04)			
T1	0.95 (0.15)	0.95 (0.19)	.54	.46	.35
T2	0.97 (0.11)	0.98 (0.04)			

Note. ACC, accuracy; RT, reaction time.

Table 3. Summary of Post Hoc Analysis of Generalized Estimated Equation Analysis

Measurement	Exercise			Control			Exercise - Control		
	Mean(95% CI)	*P* value	SMD	Mean(95% CI)	*P* value	SMD	Adjusted additional change difference (95% CI)	*P* value	SMD
**Primary outcomes**									
Internalizing problems									
Depression									
T1-T0	-2.57 (-3.76 to -1.37)	< .001	-0.71	-0.05 (-0.64 to 0.55)	.87	-0.01	-2.52 (-3.84 to -1.19)	< .001	-0.59
T2-T0	-2.30 (-3.44 to -1.17)	< .001	-0.64	0.29 (-0.35 to 0.92)	.37	0.05	-2.59 (-3.88 to -1.30)	< .001	-0.61
Anxiety									
T1-T0	-2.91 (-3.94 to -1.89)	< .001	-0.88	0.43 (-0.25 to 1.11)	.21	0.08	-3.32 (-4.56 to -2.12)	< .001	-0.77
T2-T0	-2.65 (-3.90 to -1.41)	< .001	-0.80	0.76 (0.08 to 1.45)	.03	0.13	-3.41 (-4.82 to -2.01)	< .001	-0.79
Stress									
T1-T0	-3.35 (-4.35 to -2.35)	< .001	-1.16	-0.91 (-1.88 to 0.08)	.07	-0.17	-2.44 (-3.83 to -1.06)	< .001	-0.62
T2-T0	-2.83 (-3.38 to -1.82)	< .001	-0.98	-0.38 (-1.70 to 0.94)	.56	-0.07	-2.45 (-4.08 to -0.81)	.003	-0.62
**Secondary outcomes**									
Psychological well-being									
Resilience									
T1-T0	7.04 (2.86 to 11.2)	< .001	0.38	-3.10 (-6.44 to 0.25)	.06	-0.17	10.14 (4.83 to 15.45)	< .001	0.57
T2-T0	4.17 (-1.94 to 10.3)	.17	0.23	-1.62 (-4.39 to 1.15)	.24	-0.09	5.79 (-0.86 to 12.44)	.08	0.33
Cognitive function									
Inhibitory control									
Valid RT, ms									
T1-T0	-51.10 (-76.00 to -26.20)	< .001	-0.95	23.5 (-1.04 to 48.0)	.06	0.05	-74.60 (-109.14 to -39.97)	< .001	-0.26
T2-T0	-44.60 (-62.22 to -22.00)	< .001	-0.83	48.1 (-3.11 to 99.3)	.06	0.10	-92.70 (-148.71 to -36.71)	.001	-0.32
Valid ACC, %									
T1-T0	0.02 (-0.00 to 0.05)	0.09	0.45	-0.06 (-0.12 to 0.00)	0.05	-0.41	0.08 (0.02 to 0.15)	0.01	0.83
T2-T0	0.01 (-0.01 to 0.03)	0.34	0.21	-0.04 (-0.10 to 0.02)	0.22	-0.26	0.05 (-0.02 to 0.11)	0.14	0.49
Invalid RT, ms									
T1-T0	-51.70 (-80.00 to -23.36)	< .001	-0.63	29.4 (-0.75 to 59.50)	.05	0.07	-81.00 (-121.98 to -40.09)	< .001	-0.30
T2-T0	-42.90 (-85.00 to -0.68)	.04	-0.52	30.6 (-16.20 to 77.50)	.19	0.07	-73.50 (-135.91 to -11.06)	.02	-0.27
Neutral RT, ms									
T1-T0	- 48.50 (-75.30 to -21.70)	< .001	-0.67	1.69 (-56.06 to 59.40)	.95	0.00	-50.16 (-113.20 to 12.87)	.12	-0.16
T2-T0	-52.50 (-83.70 to -21.30)	< .001	-0.72	45.33 (9.41 to 81.30)	.01	0.09	-97.83 (-144.96 to -50.71)	< .001	-0.32

Note. ACC, accuracy; CI, confidence interval; RT, reaction time; SMD, standardized mean difference.
